# Endothelial *Nogo-B* Suppresses Cancer Cell Proliferation via a Paracrine TGF-β/Smad Signaling

**DOI:** 10.3390/cells11193084

**Published:** 2022-09-30

**Authors:** Hengyu Li, Zhuo Cheng, Pinghua Yang, Wei Huang, Xizhou Li, Daimin Xiang, Xiaojun Wu

**Affiliations:** 1 Department of Breast and Thyroid Surgery, Changhai Hospital, Naval Military Medical University, Shanghai 200433, China; 2 Department of Oncology, Third Affiliated Hospital of Naval Military Medical University, Shanghai 200438, China; 3 Department of Hepatic Surgery, Third Affiliated Hospital of Naval Military Medical University, Shanghai 200438, China; 4 Department of Neurosurgery, The First People’s Hospital of Yunnan Province, Kunming 650032, China; 5 State Key Laboratory of Oncogenes and Related Genes, Shanghai Cancer Institute, Renji Hospital, Shanghai Jiao Tong University School of Medicine, Shanghai 200127, China; 6 Department of Neurosurgery, Fudan University Shanghai Cancer Center, Shanghai 200032, China; 7 Department of Oncology, Shanghai Medical College, Fudan University, Shanghai 200032, China

**Keywords:** endothelial cells, *Nogo-B*, hepatocellular carcinoma, glioma, TGF-β

## Abstract

*Nogo-B* has been reported to play a critical role in angiogenesis and the repair of damaged blood vessels; however, its role in the tumor microenvironment remains unclear. Here, we observed the differential expression of *Nogo-B* in endothelial cells from hepatocellular carcinoma (HCC) and glioma samples. Downregulation of *Nogo-B* expression correlated with the malignant phenotype of cancer and a poor prognosis for patients. In subsequent studies, endothelial *Nogo-B* inhibition robustly promoted the growth of HCC or glioma xenografts in nude mice. Intriguingly, endothelial *Nogo-B* silencing dramatically suppressed endothelial cell expansion and tumor angiogenesis, but potently enhanced the proliferation of neighboring HCC and glioma cells. Based on the results of the ELISA assay, *Nogo-B* silencing reduced TGF-β production in endothelial cells, which attenuated the phosphorylation and nuclear translocation of Smad in neighboring cancer cells. The endothelial *Nogo-B* silencing-mediated increase in cancer cell proliferation was abolished by either a TGF-β neutralizing antibody or TGF-β receptor inhibitor, indicating the essential role for TGF-β in endothelial *Nogo-B*-mediated suppression of cancer growth. These findings not only broaden our understanding of the crosstalk between cancer cells and endothelial cells but also provide a novel prognostic biomarker and a therapeutic target for cancer treatments.

## 1. Introduction

Angiogenesis is the development of new blood vessels from pre-existing vessels; this process is required for embryogenesis, normal tissue development, wound healing and tumor growth [1]. However, based on accumulating evidence, vascular endothelial cells in tumors are different from normal endothelial cells [2]. Croix et al. revealed the altered gene expression pattern in tumor endothelial cells compared to normal endothelial cells [3]. The two cell types may also respond differently to epidermal growth factor (EGF), adrenomedullin and VEGF from the microenvironment [4,5]. Moreover, tumor endothelial cells directly regulate the proliferation of cancer cells [6,7], suggesting that tumor blood vessels might have other unexpected roles within the bulk tumor.

Nogo isoform-B (*Nogo-B*), also known as reticulon 4B, is a member of the reticulon family [8]. *Nogo-B* is expressed at high levels in the microvessels [9]. In a recent study by Wälchli et al., another isoform of Nogo, Nogo-A, functioned as a negative regulator of angiogenesis in the developing central nervous system [10]. *Nogo-B* knockout (*Nogo-B^−/−^*) mice exhibit impaired arteriogenesis, indicating an essential role for *Nogo-B* in physiological angiogenesis [9]. *Nogo-B* knockout animals exhibit excessive repair of the intimal and medial layers of the balloon traction-injured femoral artery, which was remedied upon the restoration of *Nogo-B* expression, suggesting a critical role for *Nogo-B* in vascular remodeling [11,12]. Nevertheless, the role of vascular endothelial *Nogo-B* in cancer development largely remains unknown.

Hepatocellular carcinoma (HCC) is the second leading cause of cancer-related mortality globally; nearly half of patients diagnosed with HCC reside in China [13]. Although remarkable progress has been achieved in understanding the molecular mechanism and developing comprehensive therapies for HCC, rendering incurable HCC curable in certain patients, the prognosis of patients with HCC remains largely disappointing [14]. *Nogo-B* was reported to be preferentially expressed in non-parenchymal cells in the liver, and upregulation of *Nogo-B* expression has been detected in patients’ cirrhotic liver tissues [15]. *Nogo-B* is potentially involved in the activation of hepatic stellate cells (HSCs), as knockdown of *Nogo-B* in HSCs dramatically impaired HSC activation [15]. Nonetheless, the role of vascular endothelial *Nogo-B* in HCC progression remains unclear. In this study, we examined *Nogo-B* expression in vascular endothelial cells in HCC tissues from patients and investigated the role of endothelial *Nogo-B* in HCC growth. Moreover, recent studies provide additional evidence that neurovascular crosstalk is more important for understanding the molecular basis of neurological diseases than originally anticipated [16,17]. Here, we also confirmed our observations in a glioma model to expand our findings to another cancer type.

## 2. Materials and Methods

### 2.1. Patients and Tumor Specimens

HCC tissue specimens (*n* = 167) were acquired from patients who underwent surgical resection at Eastern Hepatobiliary Surgery Hospital (EHBH) in Shanghai between January 2000 and December 2009. The demographic and baseline characteristics of the patients are shown in Supplementary Table S1. Patients were followed at clinical visits every 2 months during the first postoperative year and at least every 4 months thereafter. Each visit included standard liver function and hematologic tests, as well as liver ultrasonography. Patients with a progressive increase in serum α-fetoprotein (AFP) levels and/or ultrasonographic detection of a new hepatic lesion were hospitalized for confirmation of the diagnosis and appropriate management. Ten fresh HCC specimens were obtained from patients undergoing hepatectomy. The acquisition of tissue specimens was approved by the Ethics Committee at Eastern Hepatobiliary Surgery Hospital and was performed in accordance with institutional and state regulations (EHBHKY2020-K-016).

### 2.2. Laser Capture Microdissection (LCM)

Tissue sectioning and staining were performed as previously described [18]. LCM was performed using the Leica Laser Capture Microdissection System (Leica AS LMD, Wetzlar, Germany). Three to four 10-µm sections from each specimen were used to capture approximately 10,000 cells (approximately 4 mm^2^). The time between when the tissue sections were removed from xylene to the completion of LCM and initiation of the RNA extraction process was limited to less than 45 min.

### 2.3. Quantitative Real-Time PCR

Total cellular RNA was isolated from cells using the RNeasy Micro kit (Qiagen, Germantown, MD, USA). First strand cDNA synthesis was performed using the Reverse Transcription System (Promega, Madison, WI, USA). Quantitative real-time PCR was performed using a SYBR Green PCR Kit (Applied Biosystems, Foster City, CA, USA) and the ViiA 7 Dx Real-Time PCR system (Applied Biosystems, Foster City, CA, USA). The sequences of the primers used in this study are: *Nogo-B*-F:5′-GCAGTGTTGATGTGGGTATTT-3′; *Nogo-B*-R:5′-CTGTGCCTGATGCCGTTC-3′; TGFβ1-F:5′-GTACCTGAACCCGTGTTGCT-3′; TGFβ1-R:5′-TGAACCCGTTGATGTCCACT-3′; β-actin-F:5′-AATCGTGCGTGACATTAAGGAG-3′; β-actin-R: 5′-ACTGTGTTGGCGTACAGGTCTT-3′. Each reaction was performed in triplicate.

### 2.4. Immunohistochemistry and Microarray Analysis

Immunohistochemistry of tumor sections was performed as previously described [18]. Primary antibodies against the following proteins were used: CD31, 1:100 dilution (Santa Cruz Biotechnology, Heidelberg, Germany), *Nogo-B*, 1:100 dilution (Abclonal, Wuhan, China) and Ki-67, 1:100 dilution (Santa Cruz Biotechnology, Heidelberg, Germany). We used serial sections of HCC tissues for microarray chips in which one section was stained with the anti-CD31 antibody and another section was stained with the anti-*Nogo-B* antibody. Then, the expression of *Nogo-B* in CD31-positive vascular endothelial cells was compared. All sections displaying immunohistochemical staining were observed and measured under an Olympus microscope (IX-70 OLYMPUS, Tokyo, Japan). The integrated optical density (IOD) was measured and calculated as IOD/total area of each image [19]. High *Nogo-B* expression is defined as sections in which the signal for positive staining was higher than the median value.

### 2.5. Lentivirus and Cell Lines

Human HCC SMMC-7721 cells, glioma U251 cells and human umbilical vein endothelial cells (HUVECs) were obtained from the American Type Culture Collection (Manassas, VA, USA) and cultured in DMEM with 10% fetal bovine serum (FBS) at 37 °C in a humidified incubator containing 5% CO_2_. HUVECs were infected with a lentivirus expressing a short hairpin RNA targeting *Nogo-B* (shRNA, target sequence: TATATCTGAGGAGTTGGT) or scrambled control, and stable transfectants were established and termed EC_shNogo-B and EC_NC, respectively. HUVECs were infected with a lentivirus expressing *Nogo-B* or GFP, and stable transfectants were established and named EC_GFP and EC_Nogo-B, respectively. All lentiviruses were purchased from Cyagen Biosciences Inc., Guangzhou, China.

### 2.6. Cell Proliferation Analysis

In total, 3 × 10^3^ cells were cultured in each well of 96-well plates in 10% FBS/DMEM. ATP activity was measured using a Cell Counting Kit-8 (CCK-8) and a Synergy 2 microplate reader at the indicated time points. The results are presented as a proliferation index relative to control cells.

### 2.7. In Vitro Co-Culture Assays

SMCC-7721 or U251 cells were mixed with 1 × 10^3^ CFSE-labeled EC_NC or EC_shNogo-B, respectively, and then seeded in 96-well plates. After a 3-day incubation, the number of cancer cells and HUVECs was counted by flow cytometry.

EC_NC and EC_shNogo-B were seeded in 15-cm Petri dishes at a density of 1 × 10^6^ cells per dish. Upon reaching 80% confluency, the medium was discarded, the monolayer was washed thrice with PBS and then media were replenished with serum-free DMEM. After a 24-h incubation, the medium was collected and filtered (0.45 µm). SMCC-7721 cells cultured in 6-well plates or 96-well plates were incubated with normal DMEM containing 10% FBS or the culture medium from EC_NC and EC_shNogo-B before analysis using a CCK-8 assay or flow cytometry assay.

### 2.8. In Vivo Co-Culture Assays

SMMC-7721 or U251 cells (1 × 10^6^) were mixed with EC_NC or EC_shNogo-B at a ratio of 1:1 and subcutaneously implanted into nude mice. Tumor weights and volumes were calculated using previously described methods [20]. For immunofluorescence staining, frozen sections of xenograft tumors were incubated with a rabbit anti-phospho-Smad2 antibody (1:100, Abcam, Cambridge, UK), followed by an incubation with Alexa Fluor 488-conjugated anti-mouse IgG and Alexa Fluor 555-conjugated anti-rabbit IgG antibodies (Invitrogen, Carlsbad, CA, USA). Nuclei were stained with 4,6-diamidino-2-phenylindole (DAPI). All animal experiments met the requirement of the Second Military Medical University Animal Care Facility and followed the USA National Institutes of Health guidelines.

### 2.9. Antibody Arrays

Soluble proteins in the medium of the stromal cell lines were measured using the Human Cytokine Array G4000 (RayBio, AAH-CYT-G4000-8, Guangzhou, China) and a Biotin Label-Based Human Antibody Array (RayBio, AAH-BLG-1-4, Guangzhou, China), each of which is capable of detecting 507 proteins. FBS-free DMEM tissue culture media were collected from 90% confluent HUVECs and filtered. Array chips treated with a serum-free medium were used for normalization. Hybridization was conducted overnight at 4 °C. All slides were scanned using a GenePix 4000B Microarray Scanner (Axon, Seattle, WA, USA) and analyzed using GenePix Pro 6.0 software (Axon, Seattle, WA, USA). The F532 median 2B532 score was used and averaged across triplicates on each array. The results were then normalized to internal controls. The KEGG analysis was performed using tools at https://david.ncifcrf.gov/ (accessed on 3 May 2016).

### 2.10. Luciferase Reporter Assays and ELISA

SMMC-7721 or U251 cells were incubated with the tissue culture media from EC_NC or EC_shNogo-B or serum-free DMEM for 48 h. SMMC-7721 or U251 cells were then transfected with the plasmids pGL-TGF-β-luc, pGL-STAT3-luc or pGL-AP-1-luc using jetPEI (Polyplus-Transfection, New York, NY, USA). Luciferase activities were measured using the Dual Luciferase Reporter Assay System (Promega, Madison, WI, USA). Luciferase activity was normalized to the activity of the Renilla luciferase control. All experiments were performed in triplicate.

TGF-β contents in the tissue culture media of EC_NC or EC_shNogo-B were measured using a commercially available kit (eBioscience, San Diego, CA, USA). The optical density was determined at 450 nm using a microplate reader. 

### 2.11. Western Blot Assays

Cells were directly lysed in Laemmli buffer. The immunoblotting procedure was performed as previously described [21]. Twenty micrograms of lysate were loaded in each well. The following antibodies were used for the procedure: *Nogo-B* (1:1000, Abclonal, Wuhan, China), p-Smad3 (1:1000, Cell Signaling Technology, Danvers, MA, USA), Smad3 (1:1000, Cell Signaling Technology, Danvers, MA, USA) and GAPDH (1:5000, Santa Cruz Biotechnology, Heidelberg, Germany). Densitometric analyses were performed using the Quantity One analysis software (Bio-Rad, Berkeley, CA, USA). The expression of these proteins was normalized to GAPDH.

### 2.12. Matrigel Tube Formation Assays

For Matrigel™ tube formation assays, 96-well plates were coated with Matrigel (BD Biosciences, Heidelberg, Germany). EC_NC or EC_shNogo-B or untransfected HUVECs were seeded on a layer of previously polymerized and growth factor-reduced Matrigel™. After an 8-h incubation, Cellomics Cytoskeletal Rearrangement Kits (Thermoscientific, Millersburg, PA, USA) were used to stain the tubes, and photomicrographs of each well were captured using an Arrayscan HCS Reader (Thermoscientific, Millersburg, PA, USA). The number and line length of the circular tubules formed by the cells were calculated using the Image-Pro^®^ Plus 4.5 software (Media CyberMetics, Rockville, MD, USA).

### 2.13. Scratch Wound-Healing Assay

HUVECs were seeded in a 12-well plate. Approximately 48 h later, when cells were 80% confluent, cells were incubated with serum-free DMEM overnight. A wound was generated by scraping the cell monolayer with a 10-µL pipette tip. Medium and non-adherent cells were removed, cells were washed twice with PBS and fresh media, supplemented with or without EGF (R&D Systems, Minneapolis, MN, USA), were added. Cells were permitted to migrate into the wound area for 7 days. Wound healing was photographed microscopically at days 0, 5 and 7 post-scratching (Carl Zeiss Meditec, Jena, Germany). The distance of the gap wound was measured using Photoshop software (ADOBE SYSTEMS INCORPORATED, (accessed on 15 March 2016)).

### 2.14. Transwell Migration Assays

The cell migration assay was performed in a 24-well Transwell migration chamber (BD Biosciences, Heidelberg, Germany) with polycarbonate filters of 6.5 mm in diameter and 8 µm in pore size. Approximately 2 × 10^5^ EC_NC and EC_shNogo-B were resuspended in serum-free DMEM and added to the upper chamber of the well, and DMEM supplemented with 10% FBS was added to the lower chamber. Cells were allowed to migrate for 18 h at 37 °C. Non-migrated cells were removed from the upper surface with a cotton swab. Cells that had migrated were fixed with 5% paraformaldehyde and stained with 1% crystal violet in 2% ethanol. The number of cells was counted using Image-Pro^®^ Plus 4.5 software (Media CyberMetics, Rockville, MD, USA) and expressed as the mean number of cells per field of view.

### 2.15. Statistical Analysis

Statistical analyses were performed using SPSS 20.0 for Windows (SPSS Inc., Chicago, IL, USA). Data are expressed as means ± SD. The significance of the difference in mean values between two groups was analyzed using two-tailed Student’s *t*-test. Pearson’s correlation analysis was conducted to assess correlations between two variables. Overall survival (OS) was defined as the interval from the date of surgery until death of any cause. Univariate and multivariate Cox proportional hazard regression analyses were performed to estimate crude or adjusted hazard ratios (HR) and their 95% confidence intervals (CIs). Kaplan–Meier and log-rank analyses were performed to compare OS and recurrence between subgroups. A *p*-value < 0.05 was considered statistically significant.

## 3. Results

### 3.1. Reduction of Tumor Endothelial Nogo-B Expression Predicts a Poor Prognosis for Patients

**Figure 1:**
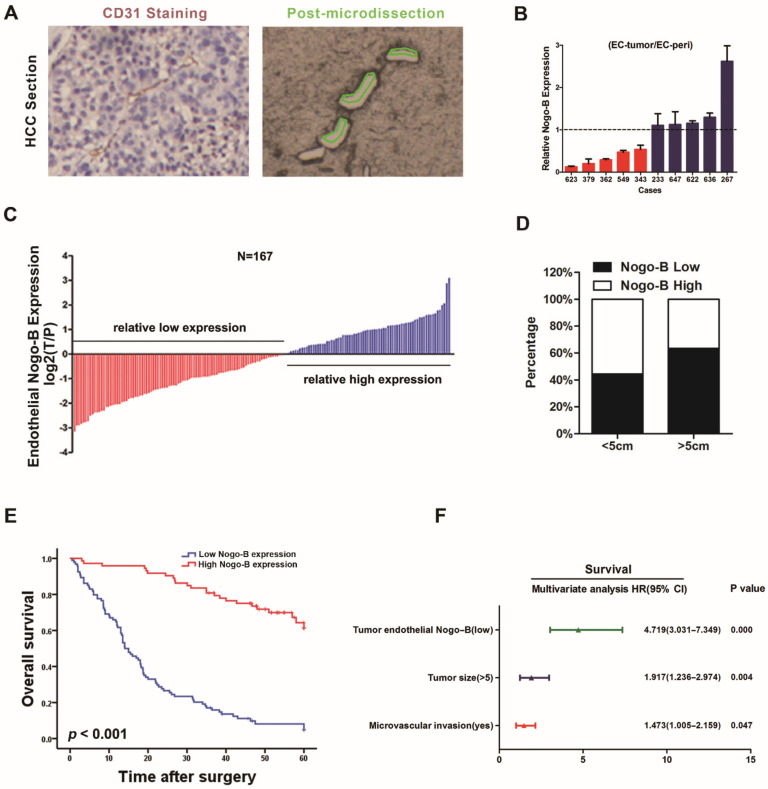
Reduced tumor endothelial *Nogo-B* expression predicts a poor prognosis for patients. (**A**) A representative image of laser capture microdissection of blood vessels. The left panel shows CD31-labeled blood vessels; the right panel shows the corresponding blood vessels in the consecutive sections that were dissected. (**B**) qRT-PCR assay of endothelial *Nogo-B* expression in the tumor and peri-tumor blood vessels obtained by laser capture microdissection from 10 patients with HCC. (**C**) Among 167 HCC specimens with paired peri-cancerous tissues, immunohistochemical staining detected lower endothelial *Nogo-B* expression in 94 cancer tissues (T) than in the corresponding peri-cancerous tissues (P), and higher endothelial *Nogo-B* expression was detected in 73 cancer tissues than in peri-cancerous tissues. (**D**) The proportion of low/high endothelial *Nogo-B* expression in patients with HCCs of sizes >5 cm/<5 cm. (**E**) Multivariate Cox regression analysis of 167 patients with HCC. *p* < 0.001. (**F**) The difference in patient survival between the high *Nogo-B* expression group and low *Nogo-B* expression group was compared using the Kaplan-Meier analysis.

We first performed double staining for CD31 and *Nogo-B* in serial sections of HCC tissues by immunohistochemistry (Supplementary Figure S1A,B) and then performed laser capture microdissection to harvest CD31-positive vascular endothelial cells from the tumor and peri-tumor tissues from 10 patients with HCC (Figure 1A) to examine *Nogo-B* expression in vascular endothelial cells from HCC. According to the results from the RT-PCR assay, half of the patients exhibited lower levels of *Nogo-B* transcripts in tumor vascular endothelial cells than in the peri-tumor vascular endothelial cells (Figure 1B). Immunohistochemistry for CD31 and *Nogo-B* in HCC tissues from 167 patients with HCC showed that 94 (56.3%) patients exhibited low *Nogo-B* expression and 73 (43.7%) patients displayed high *Nogo-B* expression in HCC endothelial cells compared with the peri-tumor endothelial cells (Figure 1C). As shown in Supplementary Table S1, reduced *Nogo-B* levels correlated with the malignant phenotype of patients’ HCC samples. Large HCC tumors (>5 cm) displayed lower levels of *Nogo-B* than small tumors (<5 cm) (Figure 1D). A significantly longer median overall survival was observed in patients with high *Nogo-B* expression than in patients with low *Nogo-B* expression (Figure 1E). Based on the results of the univariate and multivariate analyses, *Nogo-B* expression was an independent determinant of patient survival (Figure 1F and Supplementary Tables S2 and S3).

### 3.2. Down-Regulation of Endothelial Nogo-B Expression Facilitates Cancer Growth

**Figure 2:**
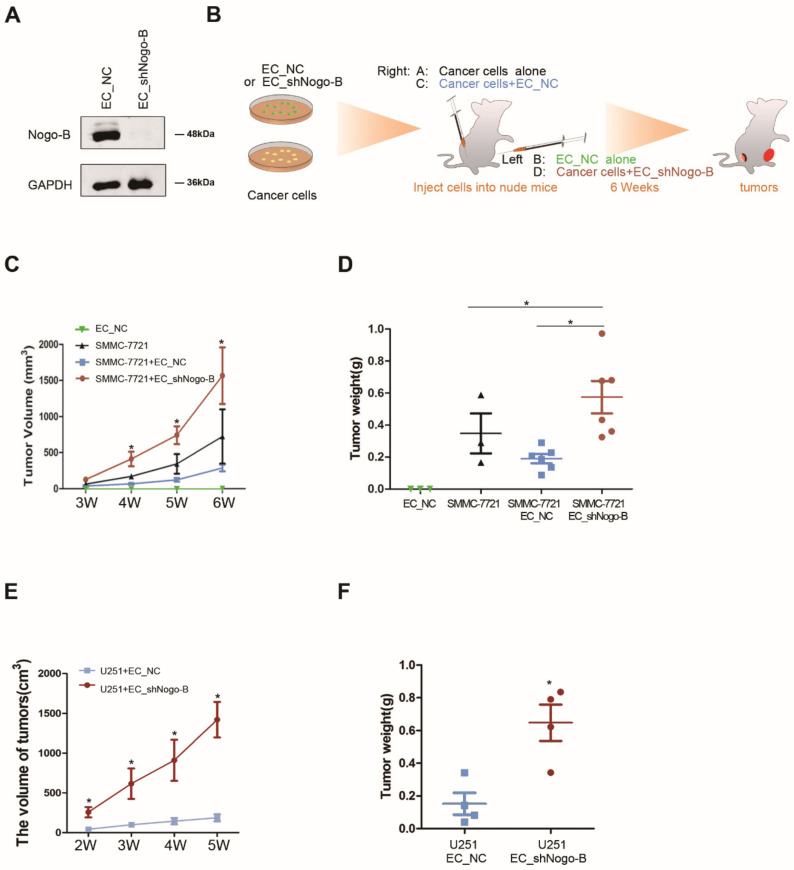
Endothelial *Nogo-B* silencing promotes tumor growth in mice. (**A**) The efficiency of shNogo-B in EC_shNogo-B was revealed by Western blot assays. (**B**) Schematic of the subcutaneous implantation of distinct cells and cell mixtures in nude mice. (**C**,**E**)The size of xenografted tumor was measured every week and the volume was calculated. “*” indicates *p* < 0.05. (**D**,**F**) Tumor weight was measured after the sacrifice of nude mice. “*” indicates *p* < 0.05.

HUVECs stably transfected with a lentivirus carrying shNogo-B (EC_shNogo-B) were established to delineate the role of endothelial *Nogo-B* in tumor growth (Figure 2A). Nude mice were subcutaneously injected with a mixed population of human ECs and SMMC-7721 or U251 (Figure 2B). According to the results of the xenograft study, mouse xenografts derived from SMMC-7721 cells and EC_shNogo-B exhibited a notable increase in both tumor volume and tumor weight compared to mouse xenografts derived from the mixture of SMMC-7721 cells and HUVECs (EC_NC) at week 6 after tumor cell implantation (Figure 2C,D). Consistent with these findings, mouse xenografts derived from glioma U251 cells and EC_shNogo-B had a greater tumor volume and tumor weight than mouse xenografts derived from U251 cells and EC_NC at week 6 after tumor cell implantation (Figure 2E,F). Moreover, C57BL/6 mice were subcutaneously injected with a mixed population of mice vascular endothelial cell C166 and mice hepatoma cell line hepa1-6. According to the results of the xenograft study, mouse xenografts derived from hepa1-6 cells and C166_shNogo-B exhibited a notable increase in both tumor volume and tumor weight compared to mouse xenografts derived from the mixture of hepa1-6 cells and C166_NC at week 4 after tumor cell implantation (Supplementary Figure S2). These findings suggest an inhibitory role for endothelial *Nogo-B* in cancer growth.

### 3.3. Endothelial Nogo-B Knockdown Impairs Angiogenesis In Vitro and In Vivo

**Figure 3:**
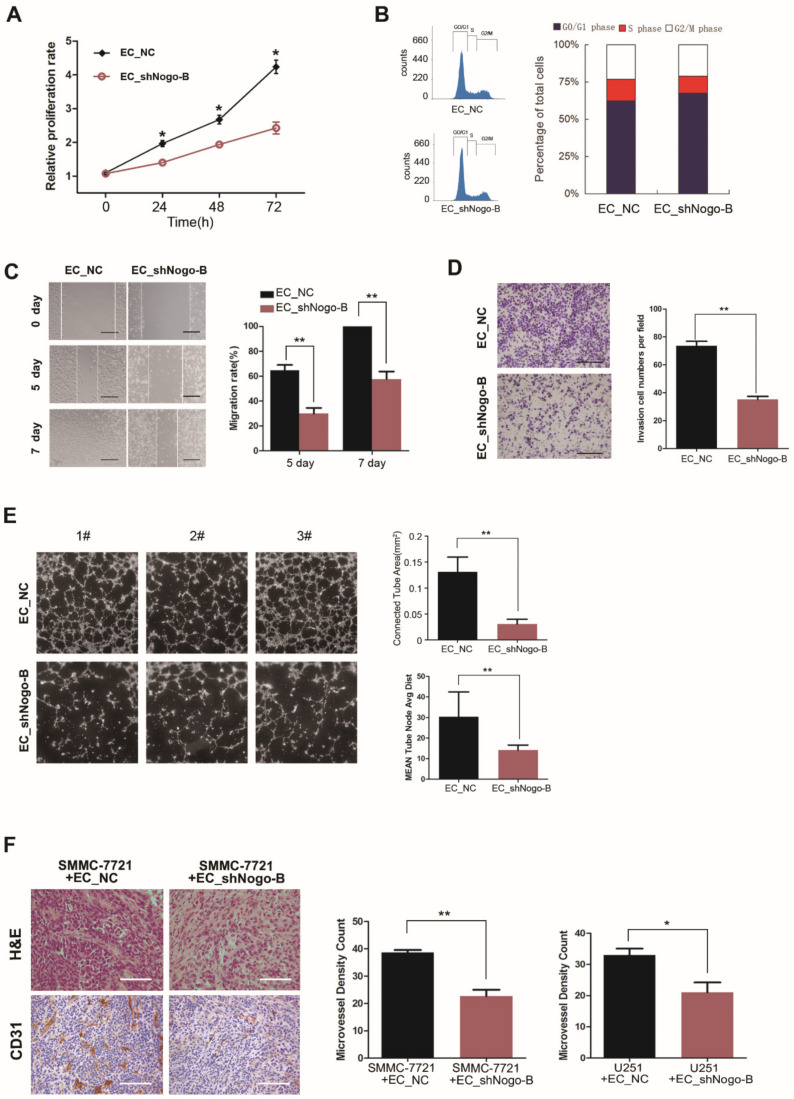
Endothelial *Nogo-B* promotes angiogenesis. (**A**) The proliferation of EC_NC and EC_shNogo-B was compared using the CCK8 assay. “*” indicates *p* < 0.05. (**B**) The cell cycle distributions of EC_NC and EC_shNogo-B were analyzed by flow cytometry, and the proportion of EC_shNogo-B in S-phase was significantly lower than EC_NC. (**C**) The migration abilities of EC_NC and EC_shNogo-B were compared using the wound healing assay. Black scale bars, 50 μm. “**” indicates *p* < 0.01. (**D**) A Transwell invasion assay was performed to compare the migration abilities of EC_NC and EC_shNogo-B. Black scale bars, 100 μm. “**” indicates *p* < 0.01. (**E**) A tube formation assay was conducted using EC_NC and EC_shNogo-B. The area under the vascular ring and number of nodules were compared. “**” indicates *p* < 0.01. (**F**) Immunohistochemical staining for CD31 in mouse xenografts derived from the mixture of cancer cells (SMMC-7721 or U251) and HUVECs (EC_NC or EC_shNogo-B); the microvessel density (MVD) assessment with CD31 immunohistochemical staining (100×). The mean of the 4 sections, with the highest vascular densities were selected, was used as the MVD for this tissue section. White scale bars, 100 μm. “*” indicates *p* < 0.05, “**” indicates *p* < 0.01.

We next explored the role of *Nogo-B* in endothelial cells to determine whether the suppressive effect of endothelial *Nogo-B* was due to its influence on angiogenesis. Based on the results of CCK8 assays, *Nogo-B* silencing significantly inhibited HUVEC proliferation (Figure 3A). The flow cytometry assay showed a reduced proportion of EC_shNogo-B in S phase compared to the control cells (Figure 3B). However, *Nogo-B* silencing did not affect the apoptosis of endothelial cells (Supplementary Figure S3). *Nogo-B* silencing markedly reduced HUVEC migration in the wound healing assay (Figure 3C). Additionally, transwell invasion assays showed that *Nogo-B* silencing significantly reduced the number of migrated HUVECs (Figure 3D). Endothelial cell tube formation assays further showed significant reductions in the area under the vascular ring and the number of nodules formed by EC_shNogo-B compared with control cells (Figure 3E). Interestingly, *Nogo-B* overexpression did not cause notable changes in HUVEC proliferation and migration (Supplementary Figure S4). Consistent with these findings, immunohistochemical staining for CD31 showed a lower microvascular density in mouse xenografts derived from the injection of SMMC-7721 or U251 cells together with EC_shNogo-B than in control xenografts (Figure 3F). These findings excluded the possibility that the suppressive effect of endothelial *Nogo-B* on tumor growth was due to its influence on angiogenesis.

### 3.4. Reduction of Endothelial Nogo-B Expression Enhances the Proliferation of Cancer Cells

**Figure 4:**
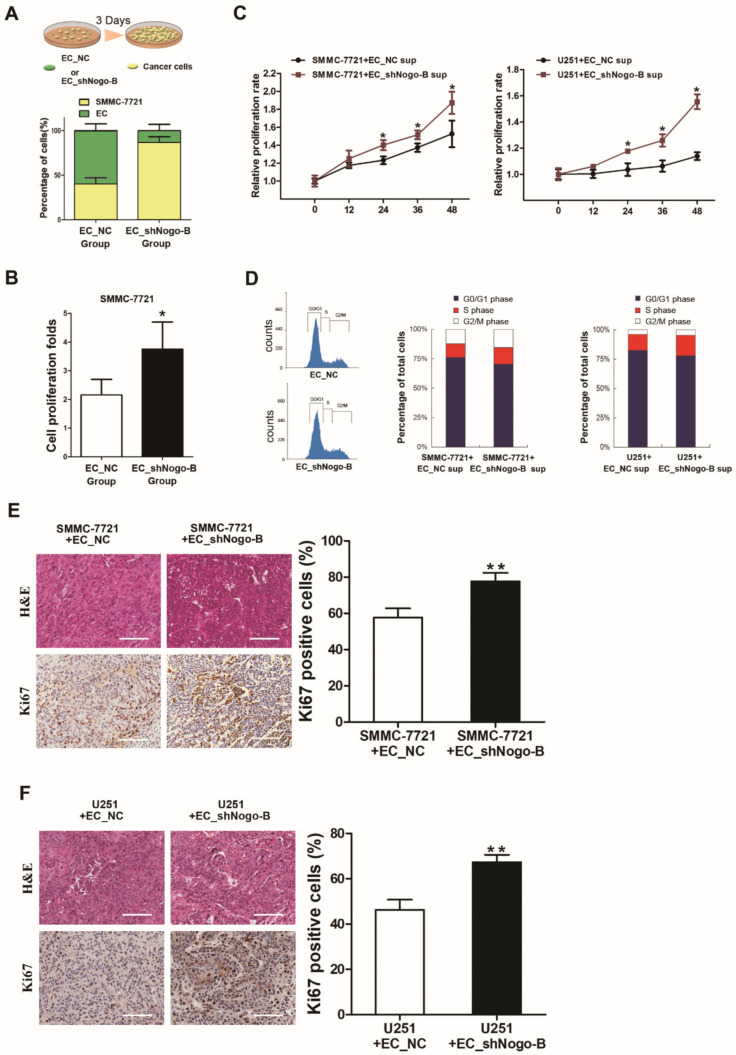
Reduced endothelial *Nogo-B* expression inhibits the proliferation of cancer cells. (**A**) SMMC-7721 cells were co-cultured with an equal number of CFSE-labeled EC_shNogo-B or EC_NC for 72 hours. The cell number was counted by flow cytometry after sorting, and the proportion of SMMC-7721 cells was calculated. (**B**) The number of SMMC-7721 cells co-cultured with EC_NC or EC_shNogo-B was counted and the fold change in proliferation was calculated after 3 days. “*” indicates *p* < 0.05. (**C**) CCK8 assays revealed that SMMC-7721 and U251 cells treated with the supernatant of EC_shNogo-B possessed a higher proliferative rate than those exposed to the supernatant of EC_NC. “*” indicates *p* < 0.05. (**D**) Flow cytometry assays revealed a higher proportion of SMMC-7721 or U251 cells in S phase after treatment with the supernatant of EC_shNogo-B than in cells exposed to the supernatant of EC_NC. (**E**,**F**) Immunohistochemical staining showed significantly higher Ki67 expression in mouse xenografts derived from the mixture of cancer cells with EC_shNogo-B than in xenografts derived from the mixture of cancer cells with EC_NC. White scale bars, 100 μm. The proportion of the Ki67 positive cells was quantified. “**” indicates *p* < 0.01.

The interplay between cancer cells and stromal cells is an important event during tumor progression. We co-cultured EC_shNogo-B or the control cells with SMMC-7721 cells to explore the possible role of endothelial *Nogo-B* in tumor cell proliferation. As shown in the cell counting data, more SMMC-7721 cells that had been co-cultured with EC_shNogo-B were observed than their counterparts that had been co-cultured with EC_NC (Figure 4A). This evident discrepancy in the number of cells was largely due to the higher proliferation rate of SMMC-7721 cells co-cultured with EC_shNogo-B than SMMC-7221 cells cultured with control cells (Figure 4B). We then collected the culture supernatants from EC_shNogo-B and EC_NC to test their effects on cancer cells. According to the CCK8 assays, SMMC-7721 cells and U251 cells treated with the supernatant of EC_shNogo-B displayed a higher proliferation rate than their counterparts that were exposed to the supernatants of control cells (Figure 4C). Consistent with these findings, flow cytometry assays revealed a higher proportion of cancer cells in S phase and a lower proportion in G0/G1 phase following treatment with the supernatant of EC_shNogo-B than for the cancer cells exposed to the supernatant of control cells (Figure 4D). The immunohistochemical staining revealed higher Ki67 expression in xenografted tumors derived from cancer cells mixed with EC_shNogo-B than in the control tumor (Figure 4E,F), further supporting the growth-inhibiting effect of endothelial *Nogo-B* on cancer cells.

### 3.5. Endothelial Nogo-B Promotes TGF-β Secretion to Inhibit Cancer Cell Proliferation

**Figure 5:**
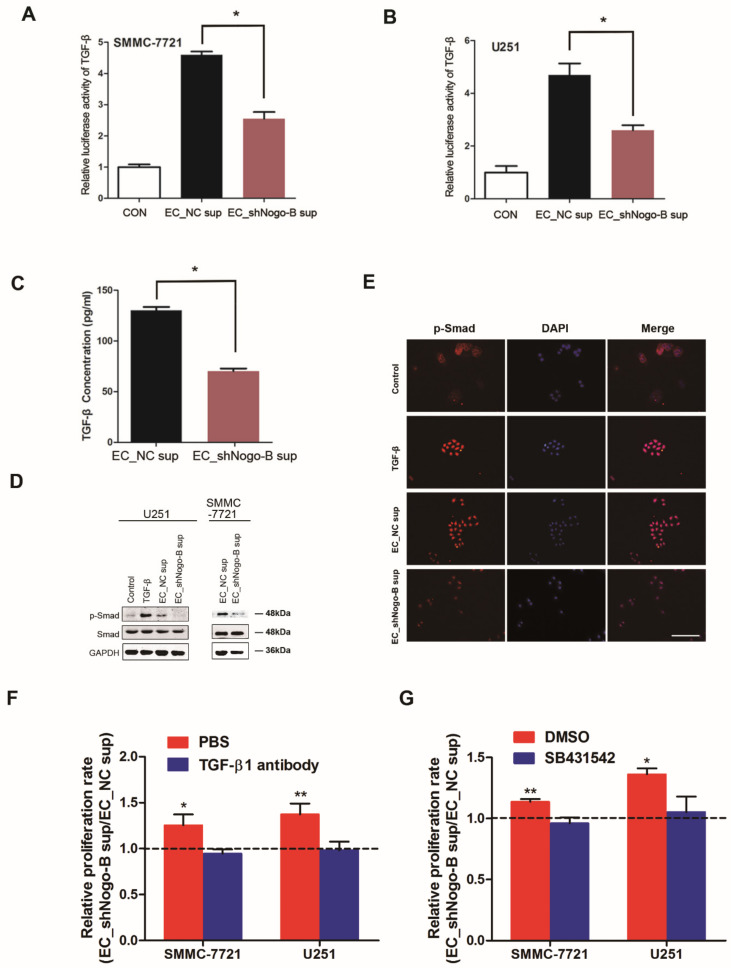
Endothelial *Nogo-B* suppresses cancer cell proliferation by promoting TGF-β secretion. (**A**) Luciferase assay of TGF-β reporter activity in SMMC-7721 cells exposed to the blank control, supernatant of EC_NC and supernatant of EC_shNogo-B. “*” indicates *p* < 0.05. (**B**) Luciferase assays of TGF-β reporter activity in U251 cells. “*” indicates *p* < 0.05. (**C**) TGF-β levels in the supernatant of EC_NC and EC_shNogo-B were measured using an ELISA. “*” indicates *p* < 0.05. (**D**) Phosphorylation of Smad3 in cancer cells treated with the supernatant of EC_NC and EC_shNogo-B was compared by Western blot assays. (**E**) Immunofluorescence staining revealed reduced nuclear translocation of phosphorylated Smad2 in SMMC-7721 cells treated with the supernatant of EC_shNogo-B compared with cells treated with the supernatant of EC_NC. White scale bars, 100 μm. (**F**,**G**) A TGF-β neutralizing antibody (0.1 µg/mL) or the Smad inhibitor SB431542 (10 µM) abrogated the difference in proliferation of cancer cells exposed to the EC_shNogo-B and EC_NC supernatants. “*” indicates *p* < 0.05, “**” indicates *p* < 0.01.

A biotin label-based human antibody array was used to compare the expression of 507 proteins in the supernatants of EC_shNogo-B and EC_NC. Differentially expressed proteins were subjected to a KEGG pathway analysis. As shown in the bioinformatics analysis presented in Supplementary Figure S5A, levels of Jak-STAT and MAPK signaling molecules were increased and levels of TGF-β signaling molecules were decreased in the supernatant of EC_shNogo-B compared to EC_NC. Nevertheless, no difference in AP-1 or STAT3 reporter activation was observed in cancer cells exposed to the supernatant of EC_shNogo-B and EC_NC (Supplementary Figure S5B), which was confirmed by Western blot assay of c-Jun or STAT3 phosphorylation (data not shown). Intriguingly, the luciferase assay showed reduced TGF-β reporter activity in cancer cells treated with the supernatant of EC_shNogo-B compared to EC_NC (Figure 5A,B). The ELISA assay revealed markedly lower TGF-β levels in the supernatant of EC_shNogo-B than in the EC_NC supernatant (Figure 5C), but no difference in the TGF-β transcript expression was detected between EC_shNogo-B and EC_NC (Supplementary Figure S5C). Western blotting assays further revealed a reduction in Smad phosphorylation in cancer cells exposed to the supernatant of EC_shNogo-B compared to EC_NC (Figure 5D). Consistent with these findings, the immunofluorescence staining revealed the decreased nuclear translocation of phosphorylated Smad in cancer cells incubated with the supernatant of EC_shNogo-B compared to EC_NC (Figure 5E). Furthermore, the EC_shNogo-B supernatant-enhanced proliferation of cancer cells was abolished by either a TGF-β neutralizing antibody or TGF-β receptor inhibitor, suggesting an essential role for TGF-β in endothelial *Nogo-B*-mediated suppression of cancer growth (Figure 5F,G). Since tumor-derived TGF-β might impact the biological properties of tumors, we detected the expression of TGF-β in cancer cells and normal endothelial cells by real-time PCR. No differences were observed among these cells (Supplementary Figure S5D).

## 4. Discussion

Angiogenesis is a critical event in cancer development due to the supply of indispensable nutrition and oxygen to tumor cells. Most of the studies related it to decreased tumor vascularization and a concomitant inhibition of tumor growth or metastasis development [22,23]. However, whether endothelial cells of blood vessels may exert other functions in the tumor mass was not well known. In the present study, *Nogo-B* was differentially expressed in the tumor vasculature, and endothelial *Nogo-B* silencing promoted endothelial cell proliferation but suppressed tumor growth via a paracrine TGFβ/Smad signaling, suggesting an extremely complex interplay between tumor blood vessels and tumor cells.

*Nogo-B* is mainly expressed in hepatic non-parenchymal cells, and its expression is upregulated in patients with cirrhosis [24]. However, the expression pattern of endothelia *Nogo-B* in cancer and its correlation with the clinical outcomes of patients remain poorly understood. The current study presents evidence that endothelial *Nogo-B* was differentially expressed in patients’ tumors. More interestingly, the median OS was significantly longer in patients with HCC who presented high endothelial *Nogo-B* expression than in patients with low endothelial *Nogo-B* levels. According to the results of our multivariate analysis, endothelial *Nogo-B* expression in the tumor is an independent prognostic determinant of patient survival. Discovery of novel biomarkers that incorporate well with traditional cancer staging may improve the prognostic predictions and beneficial effects of therapies on patients. Thus, endothelial *Nogo-B* expression in the tumor represents a potential diagnostic and therapeutic biomarker.

Angiogenesis plays an essential role in tumor growth and has become an attractive target for cancer therapy [25]. Emerging evidence has indicated a role for endothelial cells in carcinogenesis and cancer therapy [26,27]. In the present study, *Nogo-B* silencing suppressed the proliferation, migration and tube formation ability of HUVECs in vitro. Consistent with these results, endothelial *Nogo-B* silencing reduced the microvessel density in mouse xenograft tumors, suggesting a positive effect of *Nogo-B* on angiogenesis. Intriguingly, knockdown of endothelial *Nogo-B* promoted the proliferation of co-cultured cancer cells, which was validated in our in vivo xenograft study. Based on these observations, we believe that the interaction between cancer cells and vascular endothelial cells in the tumor is rather complicated. The disappointing effects of current anti-angiogenesis therapy may be at least partially due to the much more complex than anticipated effects of anti-angiogenesis treatment. In addition, anti-angiogenesis therapy usually affects the function of normal endothelial cells and sometimes interrupts physiological angiogenesis. Here, *Nogo-B* delivery did not affect the proliferation and migration of HUVECs, which express high levels of endogenous *Nogo-B*, suggesting that endothelial cell-specific *Nogo-B* delivery might be a novel anti-tumor therapy to suppress cancer growth without causing adverse effects.

The crosstalk between cancer cells and stromal cells is critical during tumor progression. Stromal cell-secreted factors have been shown to remarkably alter the characteristics of tumor cells [28]. Here, the preliminary screen using a protein array showed that *Nogo-B* silencing reduced the secretion of TGF-β signaling-associated molecules in HUVECs. In a subsequent study, we clarified that *Nogo-B* increased TGF-β production in endothelial cells. As a cytokine, TGF-β is known to maintain cell morphology and restrict cell proliferation [29]. Nevertheless, complicated and even conflicting roles of TGF-β have been observed in various tumor models. As shown in the study by Krishnan et al., TGF-β induces the expression of VEGF and placental growth factor (PlGF) under normoxic and hypoxic conditions, defining a potential indirect proangiogenic activity of TGF-β in glioblastoma [30]. In the present study, *Nogo-B*-enhanced TGF-β secretion in endothelial cells activated Smad signaling in neighboring tumor cells and, thus, suppressed tumor growth. *Nogo-B* is a component of the endoplasmic reticulum, which is required for protein synthesis. Loss of *Nogo-B* is sufficient to affect ER morphology [31], which might at least partially explain why *Nogo-B* depletion in endothelial cells reduced the TGF-β secretion. Additional mechanistic studies are warranted to delineate the detailed molecular mechanism.

In summary, our study reported the differential expression of endothelial *Nogo-B* in tumors and the correlation between endothelial *Nogo-B* expression and patient survival, providing the first evidence for the clinical significance of endothelial *Nogo-B*. Furthermore, the functional studies revealed that endothelial *Nogo-B* enhanced TGF-β secretion and, thus, suppressed cancer growth by activating Smad signaling in neighboring cancer cells (Graphical Abstract). These data further unraveled the complexity of the tumor microenvironment and raised necessary concerns regarding anti-angiogenesis therapy.

## Data Availability

Data of this study are available from the corresponding author upon reasonable request and an appropriate institutional collaboration agreement.

## Supplementary Materials

The following supporting information can be downloaded at: https://www.mdpi.com/article/10.3390/cells11193084/s1. Figure S1: Double staining for CD31 and *Nogo-B* in HCC tissues, Figure S2: Down-regulation of endothelial *Nogo-B* expression facilitates cancer growth, Figure S3: *Nogo-B* silencing did not influence the apoptosis of endothelial cells, Figure S4: *Nogo-B* overexpression did not cause notable changes in HUVEC proliferation and migration, Figure S5: Endothelial *Nogo-B* does not influence Jak-STAT and MAPK signaling. Table S1: Clinicopathological factors of 167 HCC Specimens, Table S2: Univariate Analysis of Prognostic Factors, Table S3: Multivariate Analysis of Prognostic Factors.
