# Supplementary material for: Endothelial Nogo-B Suppresses Cancer Cell Proliferation via a Paracrine TGF-β/Smad Signaling
Source: Cells. 2022 Sep 30;11(19):3084. doi: 10.3390/cells11193084 (PMC9564156; doi:10.3390/cells11193084)
Supplement: Supplementary file 1 [file cells-11-03084-s001.zip › cells-1845030-supplementary.pdf]

Endothelial Nogo-B Suppresses Cancer Cell Proliferation via a Paracrine TGF-β/Smad Signaling

Hengyu Li [1,†,*], Zhuo Cheng [2,†], Pinghua Yang [3,†], Wei Huang [4,†], Xizhou Li [1,*], Daimin Xiang [5,*] and Xiaojun Wu [6,7,*]

[1] Department of Breast and Thyroid Surgery, Changhai Hospital, Naval Military Medical University, Shanghai 200433, China
[2] Department of Oncology, Third Affiliated Hospital of Naval Military Medical University, Shanghai 200438, China; chengzhuo1219@smmu.edu.cn
[3] Department of Hepatic Surgery, Third Affiliated Hospital of Naval Military Medical University, Shanghai 200438, China; yangphua@smmu.edu.cn
[4] Department of Neurosurgery, The First People's Hospital of Yunnan Province, Kunming 650032, China; 2016122659@jou.edu.cn
[5] State Key Laboratory of Oncogenes and Related Genes, Shanghai Cancer Institute, Renji Hospital, Shanghai Jiao Tong University School of Medicine, Shanghai 200127, China
[6] Department of Neurosurgery, Fudan University Shanghai Cancer Center, Shanghai 200032, China
[7] Department of Oncology, Shanghai Medical College, Fudan University, Shanghai 200032, China
* Correspondence: lhy@smmu.edu.cn (H.L.); lixizhou@smmu.edu.cn (X.L.); xiangdaimin@sjtu.edu.cn (D.X.); wuxiaojun@shca.org.cn (X.W.)
† These authors have contributed equally to this work.

Supplementary Figures and Figure Legends

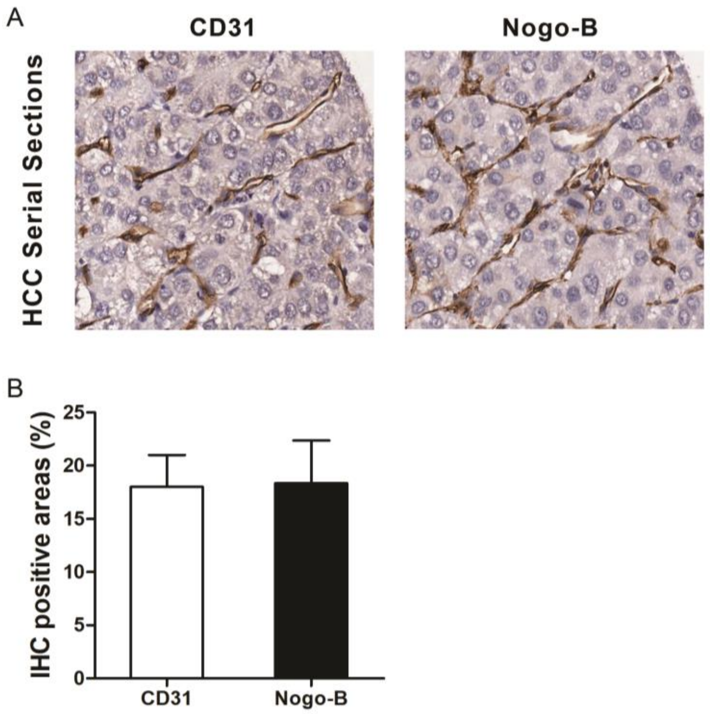

Supplementary Figure S1

(A & B) Double staining for CD31 and Nogo-B in serial HCC sections using immunohistochemistry. The proportion of the CD31 and Nogo-B positive areas was quantified.

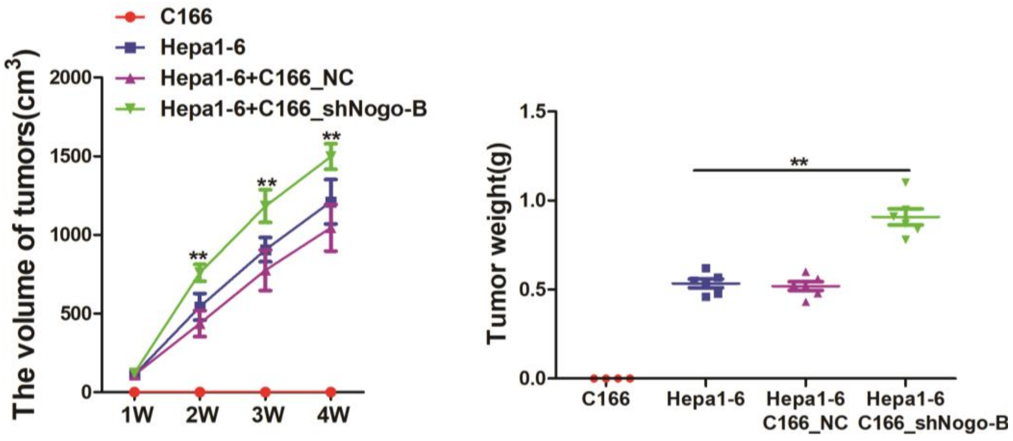

Supplementary Figure S2

The size of xenografted tumor was measured every week and the volume was calculated. Tumor weight was measured after the sacrifice of nude mice. “**” indicates *P*<0.01.

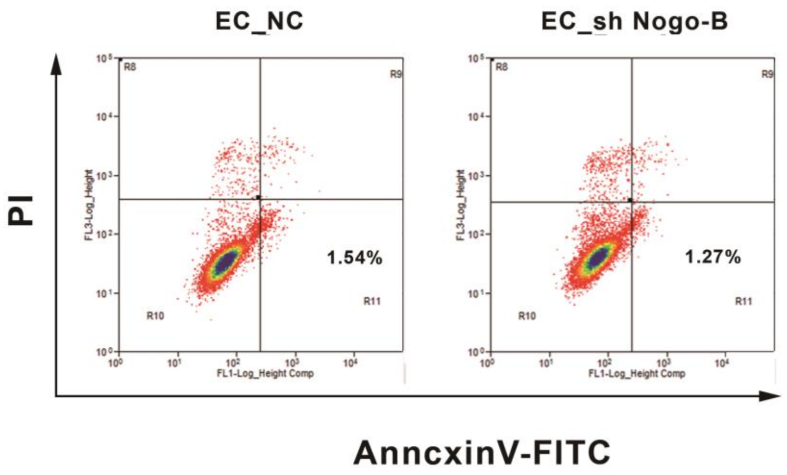

Supplementary Figure S3

The cell apoptosis of EC_NC and EC_shNogo-B was analyzed by flow cytometry, and the data showed that Nogo-B silencing did not influence the apoptosis of endothelial cells.

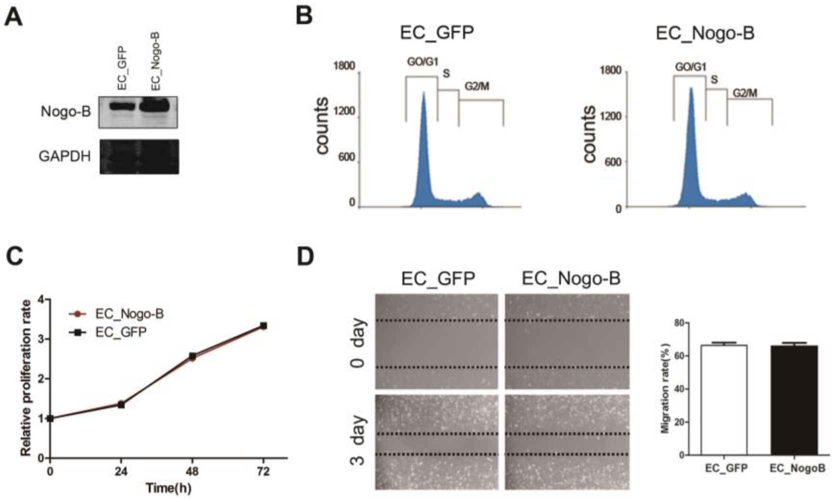

Supplementary Figure S4

(A) Overexpression of Nogo-B in EC cells was identified by western blotting.

(B) The CCK8 assay revealed that Nogo-B overexpression did not affect the cell cycle transition of HUVECs.

(C) Flow cytometry assay showing that Nogo-B overexpression did not influence HUVEC proliferation.

(D) Migration ability of ECs with or without Nogo-B overexpression was compared using the wound healing assay.

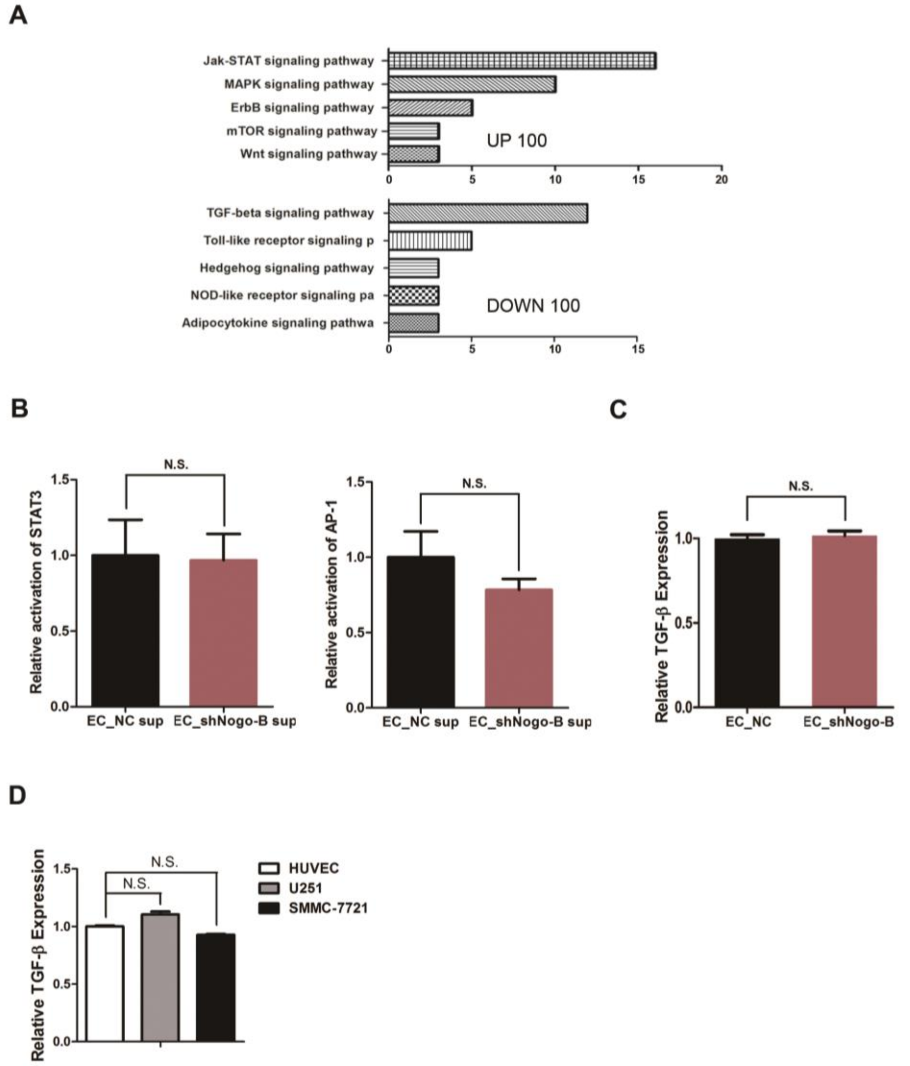

Supplementary Figure S5

(A) Significantly enriched pathways in EC_shNogo-B compared to EC_NC, which was composed of the differentially secreted proteins among the total of 507 proteins.

(B) Luciferase assay of STAT3 and AP-1 reporters in SMMC-7721 cells treated with EC_NC and EC_shNogo-B supernatants.

(C) TGF-β mRNA expression in EC_NC and EC_shNogo-B was compared using qRT-PCR assays.

(D) TGF-β mRNA expression in SMMC-7721 cells, U251 cells and HUVECs was compared using qRT-PCR assays.

Supplementary Tables

Supplementary Table S1. Clinicopathological factors of 167 HCC Specimens

Variable		Number and value(range)		*P*
		Nogo-B high expression in tumor EC(73)	Nogo-B low expression in tumor EC(94)	
**Median(range.yrs)**		49(10-74)	50(18-79)	0.614
**Gender**				
	male	59	77	0.857
	female	14	17	
**AFP(g/L)***				
	≤400	50	44	0.005
	>400	23	50	
**HBeAg**				
	positive	22	20	0.191
	nagetive	51	74	
**Tumor size(cm)***				
	≤5	35	28	0.016
	>5	38	66	
**Tumor number***				
	single	65	67	0.005
	multiple	8	27	
**Microvascular invasion**				
	yes	28	44	0.274
	no	45	50	
**Portal vein tumor thrombus**				
	yes	7	13	0.402
	no	66	81	
**Encapsulation**				
	yes	28	31	0.471
	no	45	63	
**BCLC**				
	A	61	61	0.007
	B-C	12	33	

HBeAg, Hepatitis Be antigen; AFP, α-fetoprotein; BCLC, Barcelona Clinic Liver Cancer Staging.

Supplementary Table S2. Univariate Analysis of Prognostic Factors

	Overall survival	
Variables	HR(95%CI)	p-value
Age(year), >50 vs ≤50	1.338(0.918-1.950)	0.130
Gender, male vs female	0.701(0.418-1.176)	0.179
HBeAg, positive vs negetive	0.897(0.578-1.391)	0.626
Tumor size(cm), >5 vs ≤5	2.288(1.494-3.504)	0.000
Tumor number, multiple vs single	2.086(1.358-3.205)	0.001
Encapsulation, no vs complete	0.723(0.482-1.083)	0.116
Microvascular invasion, yes vs no	1.738(1.197-2.525)	0.004
Portal vein tumor thrombus, yes vs no	2.058(1.203-3.523)	0.008
AFP(ng/mL), >400 vs ≤400	1.511(1.039-2.198)	0.031
Nogo-B, low vs high	4.854(3.125-7.519)	0.000

Supplementary Table S3. Multivariate Analysis of Prognostic Factors

	Overall survival	
Variables	HR(95%CI)	p-value
Tumor size(cm), >5 vs ≤5	1.917(1.236-2.974)	0.004
Microvascular invasion, yes vs no	1.473(1.005-2.159)	0.047
Nogo-B, low vs high	4.719(3.031-7.349)	0.000

Variables with p-value<0.05 in the univariate analysis were included in the multivariate analysis using Cox regression model.
